# Quality of Active versus Spontaneous Reporting of Adverse Drug Reactions in Pediatric Patients: Relevance for Pharmacovigilance and Knowledge in Pediatric Medical Care

**DOI:** 10.3390/ph15091148

**Published:** 2022-09-14

**Authors:** Anne T. M. Dittrich, Nori J. L. Smeets, Emma F. M. de Jong, Juliët L. Kämink, Yvet Kroeze, Jos M. Th. Draaisma, Eugène P. van Puijenbroek, D. Maroeska W. M. te Loo

**Affiliations:** 1 Department of Pediatrics, Radboud University Medical Centre, Radboud Institute for Health Sciences, Amalia Children’s Hospital, 6500 HB Nijmegen, The Netherlands; 2 Department of Pharmacology and Toxicology, Radboud University Medical Centre, Radboud Institute for Health Sciences, 6500 HB Nijmegen, The Netherlands; 3 Radboudumc Technology Center Clinical Studies, Radboudumc, 6500 HB Nijmegen, The Netherlands; 4 Netherlands Pharmacovigilance Centre Lareb, 5237 MH ‘s-Hertogenbosch, The Netherlands; 5 Unit of Pharmacotherapy, Epidemiology and Economics, Groningen Research Institute of Pharmacy, University of Groningen, 9712 CP Groningen, The Netherlands; 6 Department of Pediatric Haematology, Radboud University Medical Centre, Radboud Institute for Health Sciences, Amalia Children’s Hospital, 6500 HB Nijmegen, The Netherlands

**Keywords:** drug safety, adverse drug reactions, pharmacovigilance, active reporting ADRs, quality of ADR reports

## Abstract

For drug safety in pediatric patients, knowledge about adverse drug reactions (ADRs) is essential to balance benefits and risks, especially because of the high incidence of off-label drug use. However, underreporting of ADRs is a serious problem, leading to a deficit in knowledge affecting clinical practice. The aim of this study is to find a method by which we can improve the quantity of ADR reporting while maintaining or improving the quality of the ADR reports. This was done in several steps. First, health care providers were educated to increase awareness of ADRs. Thereafter, a novel active supporting system was introduced, where reporting ADRs was simplified; if clinical physicians suspected an ADR, they only had to send the name or hospital number of the patient, the observed ADR, and the suspected drug to a supportive team. This team collects all information needed about the possible ADR from the patient’s medical records and hospital charts. With this information, the supportive team fills in the forms necessary for reporting ADRs to the nationwide pharmacovigilance centre Lareb. With this system, the quantity of ADR reports from both inpatients and outpatients rose dramatically. Subsequently, the quality of the obtained ADR reports was measured using the ClinDoc and vigiGrade systems. This study shows there is no loss of quality of the ADR reports in the active reporting system compared to spontaneous reporting systems. Based on the data of the present study, we suggest that an active reporting system has the potential to increase our knowledge about ADRs in pediatric patients.

## 1. Introduction

Adverse drug reactions (ADRs) are a major problem in the pediatric population, leading to morbidity, mortality, unplanned or prolonged hospitalization, and increased healthcare costs [1,2]. In pediatric patients, the incidence of off-label prescribing is very high [3,4,5]. Although the correlation is not clear yet [5,6], use of off-label drugs seems to be associated with a high amount and different clinical presentation of ADRs [4]. This indicates even more that optimal recognition, treatment, and, most of all, prevention of ADRs in pediatric patients is necessary.

In order to prevent ADRs and improve drug safety, documenting evidence about ADRs is necessary in this vulnerable group of patients. Unfortunately, underreporting by health professionals is a major problem [7,8]. This may be due to lack of time, lack of knowledge on how to report [9], as well as other factors such as ignorance, indifference, insecurity, or complacency [10]. Spontaneous ADR reports coming from doctors, pharmacists, and also patients are an important source of this information after a drug has been approved for marketing [11,12]. Spontaneous ADR reporting is a useful method to get a first impression of the causal relationship between exposure to a drug and the occurrence of an ADR, but sufficient quantity and quality of reports are required.

A decrease in underreporting ADRs alone is not sufficient. If these reports lack quality, they are less useful. That is why improvement should focus on both the quantity and quality of ADR reports. Previous studies have shown that the quantity of ADR reports can be increased by training health care professionals [13,14,15], however, these studies did not systematically evaluate whether the increase in quantity also leads to a similar or even better quality of spontaneous reporting of ADRs in pediatric patients.

The aim of this study is to find a method by which we improve the quantity of ADR reporting while maintaining or improving the quality of the ADR reports. Therefore, we educated health care providers to increase awareness of ADRs and introduced a novel active supporting system to report ADRs in one of the seven academic hospitals in the Netherlands. Subsequently, the quality of the actively reported ADRs was compared with the quality of ADRs reported spontaneously at the nationwide centre for pharmacovigilance in the Netherlands (Lareb). As far as we know, this is the first study that systematically analysed the quality of reports done by both spontaneous and active reporting.

## 2. Results

### 2.1. Effect of Education on Quantity of ADR Reports

In June 2018, education was provided. In September 2018, a total of 228 children were hospitalized. At that time point, our hospital did not have an active supporting registration system for ADRs. Of the 228 patients, 221 (97%) received one or more drugs and were included in this study. In total, 101 suspected ADRs were found by the researchers. Of those suspected ADRs, 41 (40%) were not noted as such in the medical records by the physicians who cared for the patients at the time. None of the found ADRs were reported to Lareb. Ninety percent of the ADRs reported by two independent researchers were congruent. In 10% of the cases, the evaluation of a third researcher was needed. Of the suspected ADRs, 32 were related to off-label drug use, and 24 of the ADRs were serious. Sixteen of the serious ADRs were related to off-label drugs (see Table 1).

The number of identified ADRs was compared to our previous results [7]. A significant improvement in ADR recognition by the medical physicians was found (*p* = 0.026). See Table 1.

### 2.2. Active Reporting System

After introduction of the active reporting system, approximately one ADR per week was reported. After one year, 51 unique reports of suspected ADRs were reported. This number was compared to the numbers of reports Lareb received from the Amalia Children’s Hospital in the years 2016, 2017, and 2018. In these years, Lareb received eight, nine, and seven reports, respectively.

Of these 51 ADRs, 16 (31%) were related to off-label drug use, 23 (45%) were severe, and 33 (65%) were uncommon (meaning less than 1% of the people using this particular drug are likely to have the adverse reaction). Of the 51 reported ADRs, 44 (86%) were serious, uncommon, or both serious and uncommon.

All 51 forms to report the ADRs to the pharmacovigilance centre Lareb were completed by a support team. These reports were subsequently included in the next steps: quality analysis.

### 2.3. Selecting Appropriate Tools for Measuring Quality of ADR Reports

**Figure 1:**
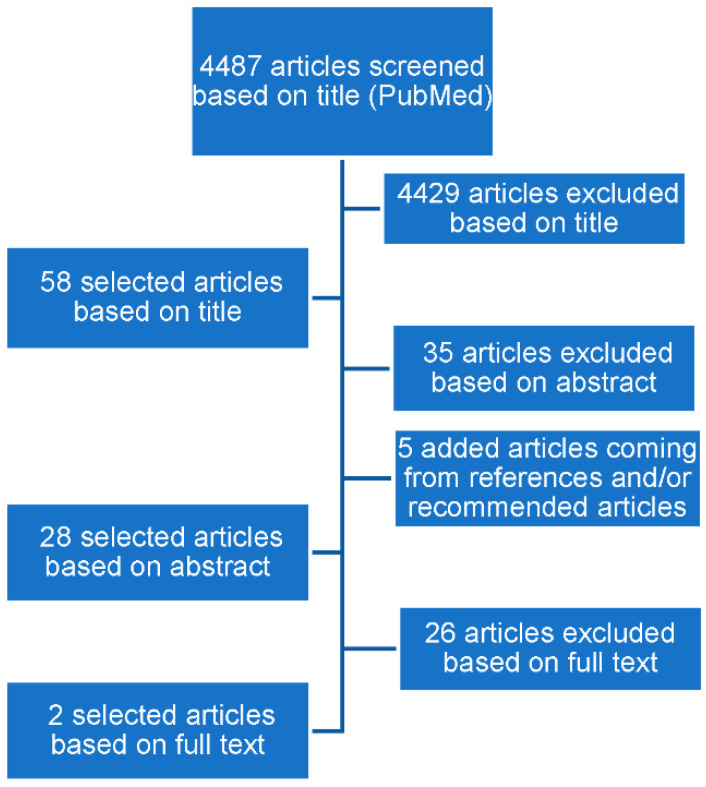
Literature search results.

The literature search resulted in 4487 articles. Two tools were selected for further analyses, as they were fulfilling the selection criteria as noted in the Methods section. The tools selected were vigiGrade [16] and ClinDoc [17] (Figure 1).

The vigiGrade tool (Appendix A) [16] measures the completeness of an ADR report on a continuous scale from zero to one. Different dimensions result in different penalties based on their relative importance for causality assessment. This results in two categories; reports with a quality of 0.8 or higher are classified as well-documented, below 0.8 are classified as not well-documented. The vigiGrade tool uses computerized software; there is no human interpretation necessary.

The second tool used is a clinical documentation tool (ClinDoc) (Appendix B) [17], which was developed by Lareb. The tool uses an approach based on the principle that completeness is not directly reflecting the amount of relevant clinical information present in a report. ClinDoc consists of four domains, including several subdomains (Appendix B). The tool is used manually and the assessor indicates which subdomains are specifically relevant for the report and then assesses whether this information is present or not. The final score is classified in one of the following three categories: excellent (≥75%), moderate (46–74%), or poor (≤45%).

### 2.4. Quality Assessment Using vigiGrade

The nationwide pharmacovigilance centre Lareb provided 162 blinded ADR reports about pediatric patients, meaning that the researchers did not know what the origin of the ADR report was. In total, 51 ADRs were from the Amalia Children’s Hospital (group A), and 111 were from other hospitals across the Netherlands (group B).

**Figure 2:**
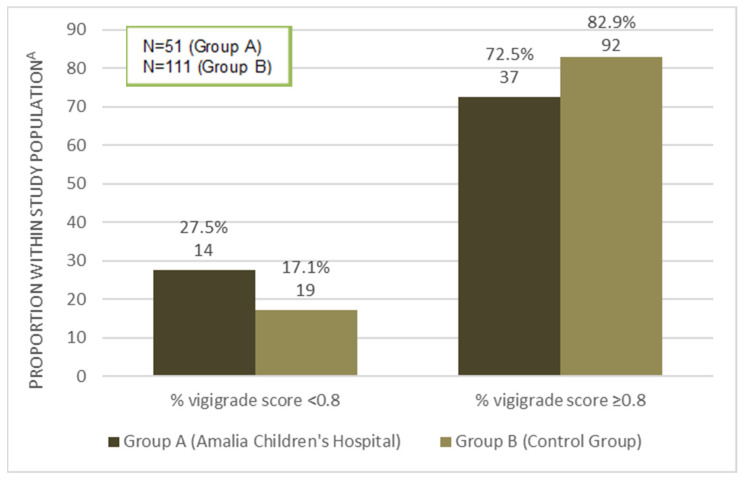
Results from vigiGrade: overview of reports in each category for Group A and Group B separately. Total percentage of reports within the two categories; the total absolute number of reports in the category is noted below the percentage.

For all reports (*n* = 162), the mean quality measured with vigiGrade was 0.90 (standard deviation (SD) 0.162). In Figure 2, the differences between groups A and B using the vigiGrade tool are presented. A well-documented report is defined as a report with total score ≥0.80 [17]. Of the 51 reports in group A, 72.5% scored ≥0.80 with vigiGrade; for the reports in group B, this number was 82.9%. This was not a statistically significant difference (Pearson Chi-square test *p* = 0.129).

### 2.5. Quality Assessment Using ClinDoc

The two researchers assessed all 162 reports separately. Thereafter, they compared their results and discussed the 23 reports that they did not score in the same category until agreement was reached. This created a third group result, further referred to as ‘consensus’. For all 23 reports, the two assessors differed only in one category. This resulted in a weighted Cohen’s Kappa of 0.68 (95% CI, 0.56 to 0.81). The mean absolute difference in total scores between these 23 reports was 15.57%.

A second measurement of ten randomly selected reports was performed to analyse the intra-assessor agreement. For assessor 1, the first and second measurement of these reports resulted in a nine out of ten match in category. For assessor 2, there was a full, ten out of ten, match in category between the two measurements. These results led to a weighted Cohen’s Kappa of 0.78 ([95% CI, 0.39 to 1.18], *p* < 0.011) for assessor 1 and a weighted Cohen’s Kappa of 1.0 for assessor 2.

**Figure 3:**
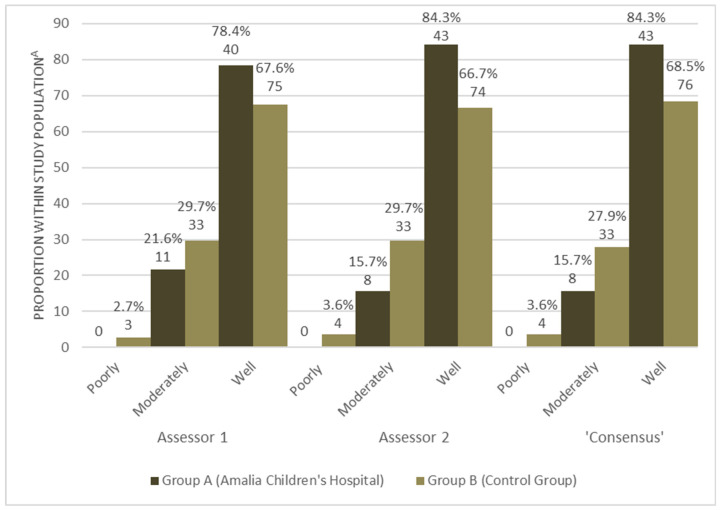
ClinDoc results: overview of the proportion of reports in each category for Group A and Group B separately. Total percentage of reports within the three categories (well, moderately, or poorly); the total absolute number of reports in the category is noted below the percentage.

The differences between group A and B were analysed. These results are presented in Figure 3. In total, 78.4% of the reports from group A scored in the category ‘well’ by assessor 1, and 84.3% of the reports scored in this category by both assessor 2 and ‘consensus’. For group B, respectively, 67.6%, 66.7%, 68.5% of the reports scored in the ‘well’ category. Of the reports in group A, 21.6% were scored in the category ‘moderately’ by assessor 1 along with 15.7% of the reports by assessor 2 and 3. In group B, 29.7% were scored in this category by both assessor 1 and 2, and this was 27.9% for ‘consensus’. Finally, all three assessors scored zero reports of group A in the category ‘poorly’, whereas 2.7% was scored in this category for group B by assessor 1 along with 3.6% by assessor 2 and ‘consensus’.

All ClinDoc scores in groups A and B were subjected to a Fisher’s exact test to analyse the differences in quality between groups A and B. For assessor 1, the test found 2.26 (*p* = 0.085). For assessor 2, the outcome was 5.553 (*p* = 0.047), and for ‘consensus’, the result was 4.663 (*p* = 0.078). All three assessments showed a trend towards higher ClinDoc scores so a higher quality of reports in favour of group A (the reports from the Amalia Children’s Hospital). Only for assessor 2 was this difference statistically significant.

## 3. Discussion

In this study, we showed that creating awareness amongst health care professionals significantly increases identification of possible ADRs. In addition, the introduction of an active reporting system increases the number of yearly reported ADRs in our hospital. Furthermore, this study shows there is no loss of quality of ADR reports in the active reporting system compared to spontaneous reporting systems.

Previous studies have shown that underreporting of suspected ADRs in a pediatric population is a serious problem [7,18]. To solve this problem is important for all patients, but especially for pediatric patients because, as highlighted before, the incidence of off-label drug use in pediatrics is very high [3,4,5]. This is associated with a high incidence of ADRs [4]. In this study we showed that, in both step 2 and 4, 31% of the ADRs we detected were related to off-label drug use. Increasing the report rate of ADRs might lead to warnings or precautions regarding particular drugs, thereby making prescribing drugs to children safer. In our mission to improve ADR reporting, we first evaluated the effect of education and creating awareness by evaluating the number of ADRs reported by clinical physicians in patients records during the period of one month. There was a difference in hospitalized patients between the two evaluated months: 301 patients in 2016 [7] versus 228 patients in the current study in 2018. The main reason for that is a change in care for pediatric oncology patients in our country. In June 2018, this care was centralized; since then, all pediatric oncology patients are admitted to a single hospital instead of all university hospitals in the country.

The effect of education and creating awareness was successful; we saw a significant increase in mentioning ADRs by medical physicians in the medical records. Of course, there might be other factors then only our actions to create awareness responsible for this increase in documented ADRs. Nevertheless, for us the possible positive effect was encouragement to continue our actions. Unfortunately, none of the suspected ADRs mentioned by medical physicians in that particular month were reported by them to the Dutch national pharmacovigilance centre Lareb, and thus knowledge regarding the ADRs that occurred remained at the hospital instead of being incorporated as knowledge for a broader audience. Therefore, based on these data, it was concluded that education and creating awareness alone is not sufficient to increase the number of reported ADRs. Subsequently, an additional step was taken to improve ADR reporting to the pharmacovigilance centre by introducing a supportive team that filled in all the forms necessary to report ADRs to Lareb, as it was suspected (based on previous research [11]) that the time needed to fill in forms was one of the major reasons ADRs were not reported to the pharmacovigilance centre.

This approach showed success; providing Lareb with 51 reports in one year, compared to only eight, nine, and seven reports in the previous years is a large difference.

This approach is comparable to the approach of Goldstein et al. [19]: they described the effect of founding an ADR network in which members of the network received a monthly email that amusingly and pleasantly reminded them to report ADRs. Members only had to make a short report and the clinical pharmacology unit completed the reports. The reporting rate increased by 80%. However, in those studies, quality or completeness of the reports was not measured, although this is essential for further analyses of the ADRs reported, because ADR reports are useless when they are lacking good quality.

Another approach to improve ADR reporting is to encourage patients and their parents to report experienced ADRs themselves. We did not look into this, but previous research showed reporting ADRs by consumers might be of great value [20], even in pediatric patients [21]. Inácio et al. stated that “patient reporting has the advantages of bringing novel information about ADRs. It provides a more detailed description of ADRs, and reports about different drugs and system organ classes when compared with health care provider (HCP) reporting. In addition, patients describe the severity and impact of ADRs on daily life, complementing information derived from HCPs” [20]. We think the same is true for pediatric patients. Most perfect would be patients and/or their parents and HCPs reporting ADRs together. This might deliver the most complete and valuable reports. This is an interesting topic for further research.

In our active reporting system, we collected on average one report per week. Keeping in mind that we identified 101 suspected ADRs with analysis of medical records in the period of one month, this number seems low. However, we noticed all reported ADRs in our active system were established ADRs, whereas the 101 found ADRs in the medical records mostly were classified as suspected. Due to the retrospective approach of the medical record analysis, we could not be sure whether the ADR was established. It makes sense that physicians only report established ADRs to the active system. One other reason for the putative low number of active reports is the fact that we asked the physicians to focus on severe and/or rare ADRs. This they did; as shown, 44 of the 51 ADR reports (86%) were serious, uncommon, or both.

As a supportive team is not directly involved in the treatment of patients that underwent ADRs, there is a risk that information sent to the pharmacovigilance centre might be incomplete or that essential information is missing. During the present study, the quality of reports performed by the supportive team was analysed and compared to reports of pediatric patients from other Dutch hospitals. In the Amalia Children’s Hospital, both academic patients and non-academic patients from our direct region are hospitalized. Therefore, we think including ADR reports from all other hospitals should be allowed. The quality assessment was done by two independent assessors who were blinded for the information about the origin of the reports. Several conclusions can be drawn from this quality assessment. First, ADR reports in The Netherlands are generally of good quality. This is not always the case, as has been reported by studies in other countries. An analysis of 7.0 million reports from VigiBase showed that only 13% of the reports have sufficient completeness (highest category) [15]. Concerning quality, Chen et al. found that only 10.18% of the 3429 Chinese ADR reports were considered to be of high quality [22]. They used a self-established reporting system for measuring report quality, which was not validated; this is the reason we did not use this system in our present study. This is comparable to a study in France that found that only 12.7% of ADR reports from general practitioners were ‘well-documented’ also using a self-established system for measuring completeness of the reports, comparable to vigiGrade [23].

Second, ADRs reported by the supportive team of the Amalia Children’s Hospital were non-inferior concerning quality of the reports, compared to ADRs reported elsewhere. Although only significant for assessor 2, there was a tendency of even a better quality in the ADRs reported by the supportive team. This is a very important finding because it shows our approach is successful, could be implemented in other hospitals, and could lead to a more rapid increase of knowledge regarding drugs used in pediatric practice. This is especially important because the majority of drugs used in pediatric practice are off-label or unlicensed [3,4,5].

This study has some important strengths. First, a control group was included. Spontaneous reporting of ADRs is the gold standard for many other countries [24]. By including these reports in the quality assessment and comparing them with ADRs reported by the supportive team, significant conclusions for clinical practice can be made. Furthermore, all analyses were done by two blinded assessors, preventing any bias in the quality assessment. Finally, two different quality tools were used to prevent bias of the tool applied, as all tools have some shortcomings. First, ClinDoc [17] is largely a subjective tool, and the subdomains could be interpreted differently by different individuals. We strived to minimalize misinterpretation by training the assessors in using the ClinDoc tool. However, along with using a subjective tool, it is inevitable that part of the inter-assessor agreement outcome is caused by the subjectivity of ClinDoc. Furthermore, some subdomains present in ClinDoc are not questioned in Lareb’s form and, in contrast, some information present in Lareb’s forms is not scored by ClinDoc. The second tool that was used, vigiGrade, is a computerized tool that scores reports based on their completeness [16]. The tool is extensive and takes many aspects of the reports into account. The vigiGrade tool uses different ‘punishments’ for lacking information based on its importance, which is a valuable strength of this tool. Another strength is that vigiGrade is a computerized tool and is therefore more objective than ClinDoc. A weakness, however, is that vigiGrade only judges the completeness of a report and does not evaluate the relevance of the clinical data in the report, unlike ClinDoc. The authors claim vigiGrade measures quality of reports; however, we think completeness of a reporting form is only part of quality measurement. We can argue that ‘the more complete the better’, and that is true, but the level of completeness does not guarantee the information is of good quality.

Although the tools we chose for this research are complementary, it is still possible that more aspects regarding quality of the ADR reports can be analysed. However, we believe this an important first step has been taken.

This study also has some limitations. First, we did not measure the level of knowledge about ADRs among our clinical physicians before starting education. This could have been useful for evaluating the quality of the education. However, this itself was not the one of the goals of this study. The second limitation is the relatively short period in which we evaluated the effect of education on ADR detection by clinical physicians. We only used one month to evaluate this, where, if we prolonged this time, we might have achieved a more accurate number of detected possible ADRs. In addition, measuring the duration of the effect of education could have been interesting. This could have been done by evaluating the number of ADRs mentioned in medical records and hospital charts two months after education was provided, then three months, four months, and so on. We would then know how often we should repeat the education to maintain the positive effect. This might be a good subject for further research. Limitations of the vigiGrade and ClinDoc tools are noted in the previous paragraph. To conclude, our data show that we were able to improve ADR reporting for pediatric patients. To achieve this on a broader scale, it is important to consider a more active, supportive reporting system in hospitals. In our hospital, we will continue with our interventions: educate the medical physicians about ADRs on a regular basis, remind them about reporting ADRs, and continue with our active reporting system in which the medical physicians still can report suspected ADRs to our supporting team. We think the effort and time investment of this team is very small and the knowledge it yields is large. Therefore, we believe this approach is sustainable for the future, although measuring sustainability was not the subject of this study.

## 4. Materials and Methods

### 4.1. Step 1 Education of Health Care Professionals, June 2018

To improve ADR reporting, the first step was to educate the clinical physicians (pediatricians, pediatric residents, and physician assistants) working at the Radboudumc Amalia Children’s Hospital. This education took one hour, and was given two times. The education included the definition of ADRs, the appearance of ADRs in the pediatric population taking the large amount of off-label prescriptions into account, causality assessment, and reporting ADRs in the Netherlands. The education was given by pediatrician–clinical pharmacologists. Primary goals of the education were to increase knowledge about ADRs in pediatric patients, increase recognition of ADRs, emphasize the importance of reporting ADRs to our national pharmacovigilance centre (Netherlands pharmacovigilance centre Lareb), and strengthen a sense of urgency to improve reporting of ADRs. To reach as many health care providers as possible, the following approaches were used: presentations (live as well as shared by email), reminders via email, and posters that were displayed at central locations in the children’s hospital. Education was provided in June 2018. In September 2018, ADRs reported in the medical records of the patients hospitalized in the pediatric wards were analysed retrospectively. They were analysed for recognizing ADRs occurring and reporting. During this month, three emails were sent to all clinical physicians to remind them about the importance of reporting ADRs. To prevent bias, the clinical physicians were not informed about the fact that analyses were performed.

### 4.2. Step 2 Measuring the Effect of Education on Quantity of ADR Reports, September 2018

The analysis was performed as described previously [7]. Briefly, medical records and hospital charts of the included patients were assessed retrospectively and manually by two independent clinical pharmacologists in training (one of them participated in the education, described in step 1). Specifically, the researchers looked for signs, symptoms, and deviating laboratory or radiology results that, in combination with the drugs the patients were using, could be considered an ADR as defined by the World Health Organization, European Medicines Agency, and the European parliament [25,26,27]. In case there was a discrepancy in assessment between the two independent researchers, a third independent researcher (an experienced clinical pharmacologist, who also was involved in the education given to the clinical physicians, described in step 1) judged the patients’ records. In addition, the seriousness of the suspected ADRs was assessed according to the EMA International Conference on Harmonization (ICH) E2A guideline [28,29]. Thereafter, relation to off-label drug use was assessed. Subsequently, the number of possible ADRs found in this project was compared to the number of ADRs reported previously [7].

### 4.3. Step 3 Active Reporting System, startin April 2019

Based on the results of step 1 and 2 (described in Section 4.1), it became clear that quantity of ADR reporting could be increased if education is given and clinical physicians are repeatedly reminded of (reporting) ADRs. Therefore, an active reporting system was introduced in our children’s hospital in April 2019. The system worked as follows: when physicians suspected an ADR, they only had to send the name or hospital number of the patient, the observed ADR, and the suspected drug to a supportive team. The supportive team consists of one pediatrician–clinical pharmacologist in training and one data manager, trained in medical data. The information could be sent by email or the internal hospital patient management system (Epic). The supportive team collected these messages, and, thereafter, collected all information needed about the possible ADR from each patient’s medical records and hospital charts. With this information, the supportive team filled in the forms necessary for reporting ADRs to the nationwide pharmacovigilance centre Lareb. These reporting forms were the same as the forms Lareb uses [30], and contained standardized questions (mandatory as well as optional) and free-text fields for additional information. The physicians were welcome to report any ADR to the supportive team, but were asked to focus on severe and/or uncommon ADRs. ADRs concerning both hospitalized patients and patients for the outpatient clinic could be reported.

### 4.4. Step 4 Evaluating Quality of ADR Reports, June 2020

**Figure 4:**
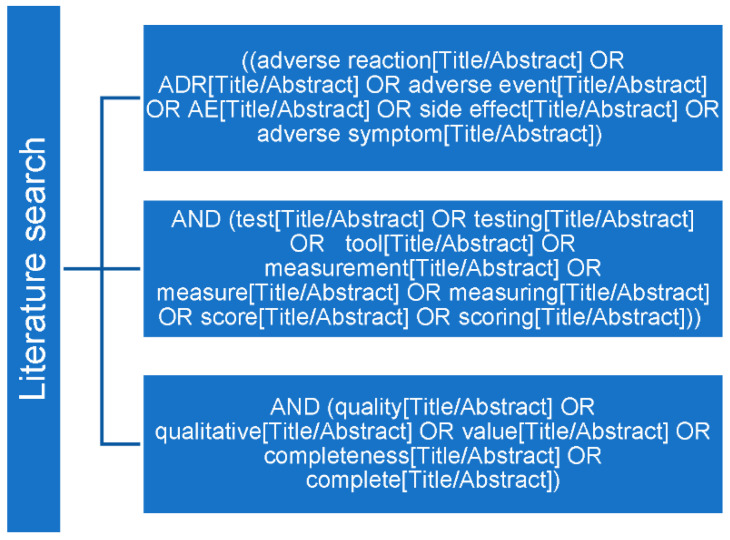
Literature search.

Approximately one year after introducing the active reporting system, the number of reported ADRs was evaluated. In addition, the relation to off-label drug use, seriousness [28,29], and rarity [31] were assessed. Subsequently, the quality of the ADR reports was analysed. Therefore, the ADR reports from the Amalia Children’s Hospital (group A) were compared to ADR reports received by Lareb concerning pediatric patients in the Netherlands (group B). To measure report quality, first an extensive literature search was performed in June 2020 in PubMed searching for appropriate tools, using the MeSH (Medical Subject Headings) terms shown in Figure 4. To be included, articles had to prescribe a validated tool for measuring quality of ADR reports and had to be written in English. There was no publication date limit. Selection of the results was based on title and abstract; the remaining articles were screened on full text.

The tools selected (vigiGrade and ClinDoc; see Results section for further information about these tools) were used according to the articles describing these tools; no adjustments were made. Assessment of the ADR reports was performed independently by two researchers who were blinded to the origin of the reports. Prior to scoring the ADR reports, both assessors were trained how to use the selected tools using five pilot reports that were not included in this study. To determine the intra-assessor reliability, ten randomly selected reports were assessed a second time by each researcher. Intra- and inter-assessor reliability were calculated using weighted Cohen’s Kappa with the following interpretation values: slight (≤0.20), fair (0.21–0.40), moderate (0.41–0.60), substantial (0.61–0.80), almost perfect (0.81–1.00) [32].

After identifying the appropriate tools to determine the quality of ADR reports, a retrospective non-randomized trial was performed to compare ADR reports from the Amalia Children’s Hospital (group A) with a control group of ADR reports concerning pediatric patients from other hospitals in The Netherlands (group B), aiming for a ratio of 1:2 (this depends on the number of reports Lareb received). All reports (groups A and B) were provided from the Lareb database, randomly ordered and anonymized. Subsequently, all information that could lead to the origin of the report was removed before they were handed to the researchers. To exclude bias, all ADR reports were matched for year of reporting, medical function of the reporter, and patient’s age.

Quality was assessed by two researchers (assessors 1 and 2) independently. Thereafter, they compared their results and discussed the reports that they did not score the same, until agreement was reached. This created a third group result, further referred to as ‘consensus’. Differences between the two groups (A and B) were calculated. This was performed three times with three different variables: the outcomes of assessor 1, assessor 2, and the consensus results (consensus).

### 4.5. Statistical Analyses

For statistical analyses, Fisher’s exact test, Pearson Chi-square test, and weighted Cohen’s Kappa were used when appropriate. A *p*-value < 0.05 was considered statistically significant. All analyses were performed using IBM SPSS Statistics, version 26 (IBM Corp: Armonk, NY, USA).

## 5. Conclusions

Clearly, the spontaneous reporting of adverse events remains essential and, as demonstrated in the present study, can be of high quality and therefore essential for increasing our knowledge. Additionally, it remains important to increase awareness and to motivate healthcare professionals to report ADRs and include all available clinical information that will contribute to proper causality assessment.

## Figures and Tables

**Table 1:** Comparison of the number of ADRs found in the two studies, showing the effect of creating awareness, number of serious ADRs, and the number of ADRs due to off-label medication.

Study	Number of Hospitalized Patients	Number of Patients with 1 or More Suspected ADRs	Total Number of Suspected ADRs	ADRs Documented by Treating Physician	ADRs Not Documented by Treating Physician	Number of Serious ADRs	ADRs Related to Off-Label Drug Use
June 2016 [7]	301	81 (26%)	132	59 (45%)	73 (55%)	16 (12%)	
September 2018	221	64 (28%)	101	60 (60%)	41 (40%)	24 (24%)	32 (31%)
*p* value				0.026			

## Data Availability

The data that support the findings of this study are available from the corresponding author upon reasonable request.

## Appendix A

Overview of the vigiGrade completeness score [16].

**Dimension**	**Description**	**Considerations**	**Penalty (%)**
**Time-to-onset**	Time from treatment start to the suspected ADR	Imprecise information penalised if there is ambiguity as to whether the drug preceded the adverse event; by 30% if the uncertainty exceeds 1 month, 10% otherwise	50
**Indication**	Indication for treatment with the drug	Penalty imposed if information is missing or cannot be mapped to standard terminologies such as ICD ^a^ or MedDRA ^b^	30
**Outcome**	Outcome of the adverse event in this patient		30
**Sex**	Patient sex	‘Unknown’ treated as missing	30
**Age**	Patient’s age at onset of suspected ADR	Age ‘unknown’ treated as missing 10% penalty imposed if only age group is specified	30
**Dose**	Dose of the drug(s)		10
**Country**	Country of origin	Supportive in causality assessment since medical practise and adverse reaction reporting vary between countries	10
**Primary reporter**	Occupation of the person who reported the case (e.g., physician, pharmacist)	Supportive in causality assessment since the interpretation of reported information may differ depending on the reporter’s qualifications	10
**Report type**	Type of report (e.g., spontaneous report, report from study, other)		10
**Comments**	Free-text information	Uninformative text snippets excluded	10
a: ICD: International Statistical Classification of Diseases and Related Health Problems; b: MedDRA: Medical Dictionary for Regulatory Activities.			

## Appendix B

Final Clinical Documentation Tool (ClinDoc) [17].

**1**	**Adverse Drug Reaction (ADR)**	**Relevant?** **Yes, No**	**Present?** **Yes, No**
A	Proper description of the ADR	Yes ^a^	
B	Specification reaction ‘localization’ and ‘characterization’ To ‘strengthen the diagnosis (subdomain c or d or e applicable):		
C	Treatment; or		
D	Visual material (photo, video); or		
E	Lab values, test		
**2**	Chronology	Relevant? Yes, no	Present? Yes, no
A	Latency	Yes ^a^	
B	Description of the course of the ADR		
C	Action taken on drug	Yes ^a^	
D	Outcome of the ADR	Yes ^a^	
**3**	Suspected drug	Relevant? Yes, no	Present? Yes, no
A	Brand name in case of drug substitution?		
B	Different forms or route of administration for suspected drug?		
C	Dose-relationship with ADR?		
D	Batch number of relevance?		
**4**	Patient characteristics	Relevant? Yes, no	Present? Yes, no
A	Risk factors/medical history/comorbidity/indication		
B	Concomitant medication	Yes ^a^	
C	Age/gender/length/weight		
D	Patient’s lifestyle or other risk factors		
a: always relevant, Calculation of score: domain score: Number of present subdomains/number of relevant subdomains * 100%, Final score: Average relevant domain scores, Cut off values: Poorly (≤45%), moderately (from 46–74%) and well (≥75%).			
